# Role of Ultrasound in Managing Cervical Polyps During Pregnancy

**DOI:** 10.7759/cureus.18702

**Published:** 2021-10-12

**Authors:** Laveena Kondagari, Lena S Josephs

**Affiliations:** 1 Obstetrics and Gynecology, NYC Health+ Hospitals/Jacobi Medical Center, Bronx, USA; 2 Obstetrics and Gynecology, Albert Einstein College of Medicine, Jacobi Medical Center, Bronx, USA

**Keywords:** antepartum hemorrhage, vaginal bleeding during pregnancy, polypectomy during pregnancy, endocervical polyp, decidual polyp, cervical polyp in pregnancy

## Abstract

No definitive management guidelines exist for cervical polyps during pregnancy. Ultrasound can aid in creating a treatment plan by assessing the type of polyp and source of symptomatology. Three pregnant patients in the first, second, and third trimesters of pregnancy presented with polyps. On examination, the polyps ranged from 2 to 6 cm in size. In all cases, the origin of the cervical polyps was first identified on ultrasound. Polypectomies were performed with no complications. All patients subsequently had uncomplicated normal spontaneous vaginal deliveries at term. Ultrasounds can help localize the source of symptomatology to polyps versus placental pathology. Additionally, ultrasound can determine the origin and type of polyp for creating an individualized, safe treatment plan during pregnancy.

## Introduction

Cervical polyps are common, benign neoplasms of reproductive-age women. They are usually diagnosed incidentally on vaginal examination and appear as soft, glistening, friable, cherry-red to reddish-purple lesions that bleed upon contact. Most cervical polyps are <2 cm, and a larger size is associated with a higher incidence of malignancy [1]. Although the exact etiology of cervical polyps is unknown, they are thought to arise secondary to reactive changes from high circulating hormone levels in cases of chronic inflammation and from the congestion of blood vessels in the cervix secondary to increased levels of estrogen or else are idiopathic in nature. They are classified according to their site of origin as endocervical polyps or decidual polyps.

Endocervical polyps originate from the endocervix and demonstrate the histological features of mucus-secreting endocervical glands with a fibrovascular stroma beneath. Decidual polyps arise from the decidua and have decidual stroma on histology. Some endocervical polyps show focal stromal pseudodecidual changes that can mimic a decidual polyp. Most polyps are asymptomatic, but some cause leukorrhea, infection, postcoital bleeding, intermenstrual bleeding, or postmenopausal bleeding. In nonpregnant patients, these polyps are removed in the outpatient setting with forceps. 

Cervical polyps are diagnosed in pregnant patients sporadically, though their exact prevalence is unknown. In pregnant patients, these polyps can be asymptomatic or else can cause recurrent vaginal bleeding, discharge, premature labor, infection, chorioamnionitis, or increased bleeding during labor. In 1.7% of pregnant and 5% of symptomatic patients, these cervical polyps were found to be precancerous or cancerous [2-3]. Regardless of whether the polyp is symptomatic or not, the diagnosis of a polyp can cause stress in pregnant patients. There are currently no definitive guidelines for when either conservative management or polypectomy should be utilized during pregnancy.

This paper describes three cases where cervical polyps were removed during the first, second, and third trimesters with no major complications. In all three cases, ultrasound was utilized to help determine the type of polyp and anticipate the prognosis of its removal. We propose a management algorithm to incorporate ultrasound in managing cervical polyps during pregnancy. Individual consents were obtained from these patients for use of their information. Institutional review board (IRB) approval was not required as per our institutional policy.

## Case presentation

Case one

**Figure 1:**
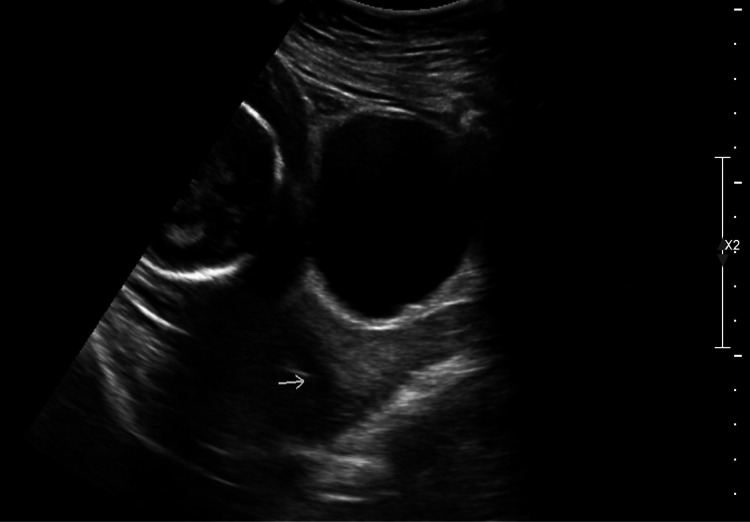
Transabdominal midline sagittal ultrasound image showing the presence of suture material in the endocervical canal after polypectomy.

A 34-year-old gravida three para two at seven weeks of gestation presented for her first prenatal care visit with intermittent spotting for two weeks and was diagnosed with a 3 cm friable endocervical polyp on speculum examination. Obstetric history included two prior normal spontaneous vaginal deliveries. Her medical history was significant for hypothyroidism treated with levothyroxine. All other causes for antepartum hemorrhage except for the presence of the polyp were ruled out. The patient was counseled about polypectomy versus observation and was scheduled for a sonogram to evaluate polyp type and rule out other causes for antepartum hemorrhage. At 14 5/7 weeks, bedside sonogram showed a 3 cm endocervical polyp with its origin near the internal opening of the cervix. The patient opted for removal of the polyp. Polypectomy was performed as detailed below with an estimated blood loss of 5 mL. Histopathology showed a 2 x 1.4 x 1 cm endocervical polyp. On an anatomy scan at 20 1/7 weeks, a suture was noted in the cervical canal (Figure 1).

During subsequent sonography in the third trimester, no suture material was found. The patient had an uncomplicated prenatal course following polypectomy. She was admitted in labor at 38 6/7 weeks and had an uneventful spontaneous vaginal delivery with an estimated blood loss of 400 mL. 

Case two

A 40-year-old gravida three para two at seven weeks and two days of gestation presented to the emergency room for nausea and vomiting and had an incidental diagnosis of 4 cm endocervical polyp on speculum examination. At 16 3/7 weeks, the patient returned to the emergency room for vaginal spotting and was discharged for outpatient follow-up. At the initial prenatal care visit, she was counseled on the options of polypectomy versus observation. Anatomy ultrasound was performed at 21 1/7 weeks gestation and was noted to have a posterior placenta, a fetus in cephalic presentation, and a 3.88 x 1.56 x 0.726 cm mass with a feeding vessel that originated in the lower part of her cervix. The polyp was removed after informed consent was obtained as detailed below. The estimated blood loss was 5 mL. Pathology showed a 2.5 x 1.7 x 0.7 cm polyp from the junctional area between the lower uterine segment and the endocervix with Arias-Stella reaction, calcification, and chronic active inflammation. She had an uncomplicated prenatal course and a normal spontaneous vaginal delivery with an estimated blood loss of 400 mL after scheduled induction of labor for advanced maternal age.

Case three

**Figure 2:**
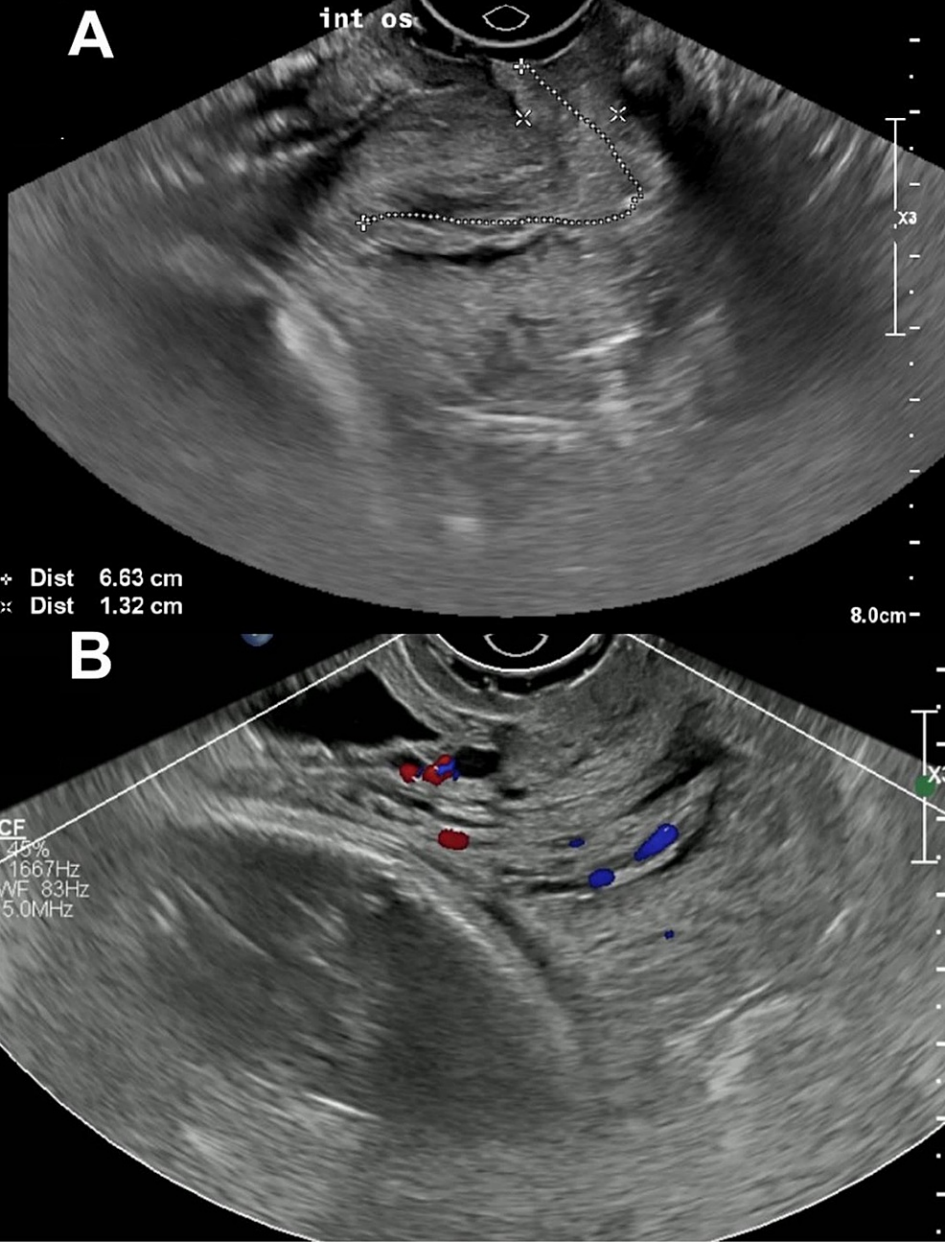
Transvaginal ultrasound. Transvaginal ultrasound showing grayscale imaging (A) and color Doppler imaging (B) of an endocervical polyp. CF: color flow, WF: wall filter, int os: internal opening of the cervix.

A 38-year-old primigravida at 32 weeks and 5 days of gestation presented to labor and delivery with heavy vaginal bleeding for three hours, cramping, and leaking of fluid. Obstetric history was significant for being a late registrant from a different country, advanced maternal age, and marginal cord insertion. On speculum examination, a 6 x 3 cm nonfriable polyp in the endocervical canal with a small amount of blood was noted in the vaginal vault. She was discharged after complete evaluation for rupture of membranes and other causes for antepartum hemorrhage. A dedicated sonogram was scheduled to evaluate the origin of the polyp. She presented to the ultrasound unit at 37 2/7 weeks actively bleeding with 50 mL of bright red blood in the vagina and was experiencing intermittent bleeding twice weekly since 35 weeks of gestation. On sonogram, a 3,105 g fetus in cephalic presentation with a posterior fundal placenta and a polyp measuring 6.63 x 1.32 x 1.68 cm, with a stalk in the cervical canal, was noted (Figure 2A, 2B).

She was transferred to labor and delivery and was counseled by maternal-fetal medicine about options to proceed with either an elective cesarean section followed by polypectomy or polypectomy and expectant management of spontaneous labor. The patient opted for the latter option. Polypectomy was performed in the operating room as described below under spinal anesthesia. Two grams of cefoxitin was given as surgical prophylaxis in anticipation of proceeding with cesarean delivery in case of prolonged bleeding after polypectomy. The polyp was friable and removed in two pieces. She was discharged home the next day. The estimated blood loss was 20 mL. Pathology showed two 2.3 x 2.0 x 0.2 cm endocervical polyps with acute and chronic inflammation, as well as microglandular hyperplasia. The patient had an uneventful prenatal course following polypectomy. She was admitted for spontaneous rupture of membranes at 39 6/7 weeks and was induced with misoprostol, Cook balloon, and oxytocin and had an uncomplicated normal spontaneous vaginal delivery with an estimated blood loss of 300 mL. 

Procedure for polyp removal

Patients were counseled about a lack of definitive recommendations in the literature regarding the management of polyps during pregnancy, and written consent was obtained after informing patients about the possible risks of bleeding, infection, preterm labor, premature rupture of membranes, chorioamnionitis, and miscarriage. All three patients had a dedicated ultrasound prior to polypectomy and were determined to have endocervical polyps with the origin in the cervical canal. Patients were kept in dorsal lithotomy position, and the perineum was prepped and draped in a normal sterile fashion. A bivalve speculum was placed in the vagina. The exact location of the polyp and stalk was then identified. The polyp was grasped with DeBakey’s forceps, and a 0-Vicryl Endoloop was used to strangulate the stalk closer to the base. The polyp was detached from its stalk using electrosurgery, and the base of the stalk was cauterized to ensure hemostasis. The fetal heart rate was assessed after the procedure by either ultrasound or performing a nonstress test.

## Discussion

There are no current management guidelines for cervical polyps during pregnancy. From the existing limited evidence, management depends on factors such as polyp type, symptoms, gestation age, prior history, and the type of operative management. In our case series, we describe three cases of cervical polypectomy from each trimester. Ultrasound was used in all cases to identify the type of polyp and counsel patients about their prognosis after polypectomy. All patients had uncomplicated normal spontaneous vaginal deliveries at term following polypectomy. 

**Figure 3:**
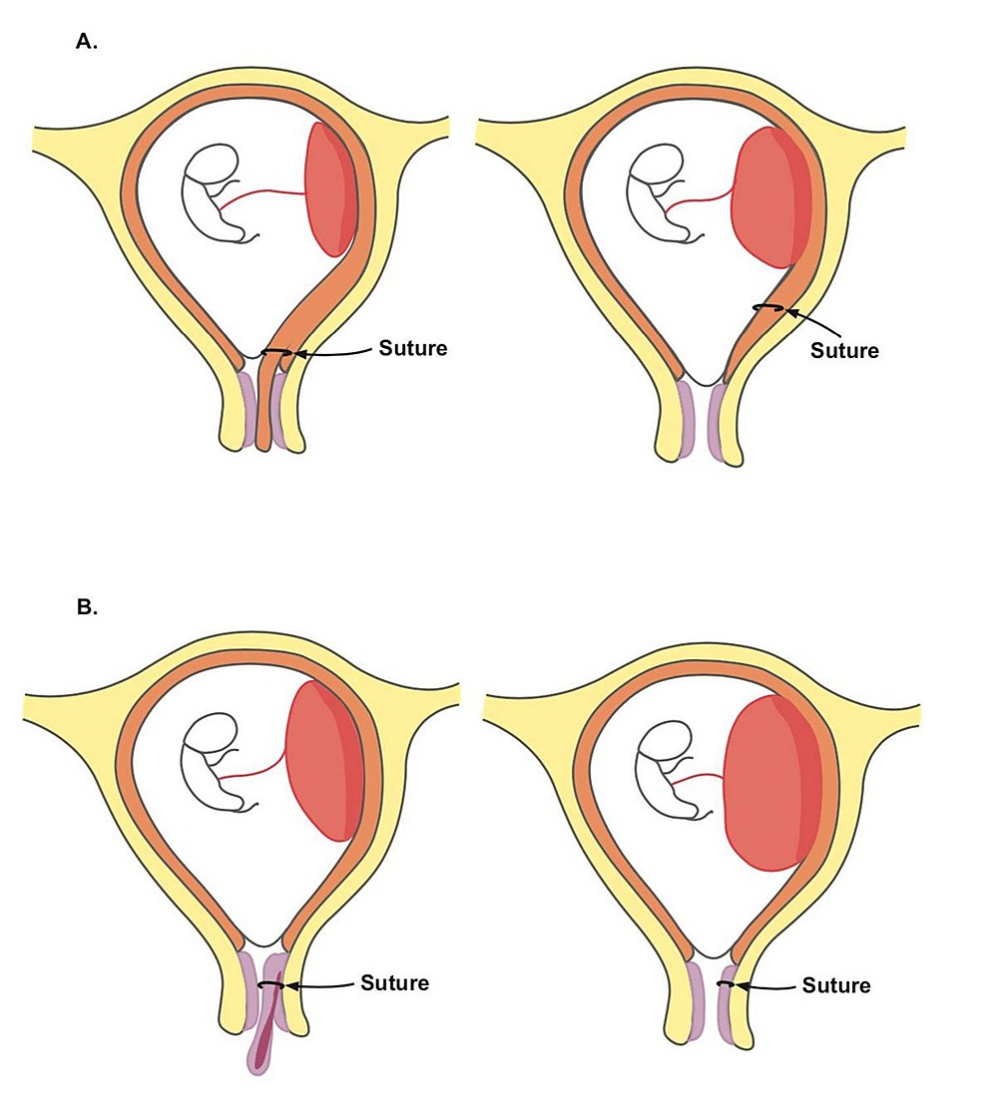
Illustration depicting suture migration. Illustration depicting suture migration after decidual polypectomy (A) compared to endocervical polypectomy (B) without suture migration.

Tokunaka et al. evinced that the patients undergoing removal of decidual polyps had a 12.2% risk of spontaneous abortion versus 0% for cervical polyps and 34.2% risk of preterm delivery for decidual polyps versus 4.8% for cervical polyps [4]. It is possible that our patients did not have complications because we were able to distinguish endocervical polyps from decidual polyps with ultrasound. On ultrasound, endocervical polyps are visualized in the endocervical canal with a feeding vessel, while decidual polyps are continuous with the decidua and reach the surface of the chorion [5-6]. Increased risk of complications on the removal of decidual polyps can be attributed to injury of the endometrium and consequently heightened risk of inflammation and infection in the uterine cavity [4]. The upward progression of decidua along with foreign suture material after decidual polypectomy might be a risk factor for ascending infection and increase the risk of complications (Figure 3A, 3B) [7].

Hence, we propose that ultrasound characteristics should be considered for risk stratification and patient counseling prior to formulating a treatment plan.

There is currently much debate over the management of cervical polyps during pregnancy. While some case reports demonstrate prolongation of the gestational period after polypectomy, others show an increased risk of miscarriage and spontaneous preterm delivery after polypectomy. The presence of polyps can alter enzymatic properties and the consistency of the cervix, resulting in increased granulocyte elastase activity and thus increased inflammation [8]. Accordingly, it is posited that the polyps can act as a nidus for infection or even result in chorioamnionitis. Studies have shown a decreased incidence of chorioamnionitis in patients who underwent polypectomy during pregnancy [8]. Conversely, there are case reports that demonstrate an increased risk of chorioamnionitis after polypectomy [7]. However, the majority of existing data does not stratify outcomes based on polyp classification. It remains to be elucidated whether the polyps themselves or the polypectomy procedures are the sources of infection. 

If resection is considered, there is a paucity of data on factors that can lead to complications. Fukuta et al. have demonstrated an increased risk of complications from polypectomy if the width of the polyp is >12 mm, if gestational age is greater than 10 weeks, or if the patient is symptomatic with vaginal bleeding [9].

**Figure 4:**
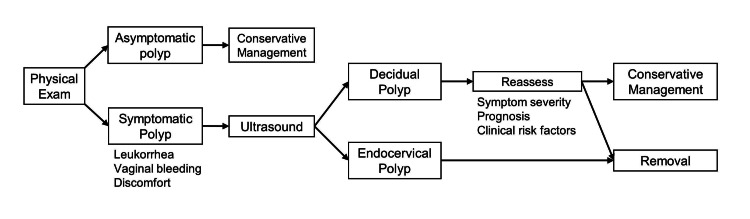
Schematic diagram representing a management algorithm for cervical polypectomy during pregnancy using ultrasound.

Due to controversial evidence and a lack of multicenter, randomized controlled trials, physicians face uncertainty when counseling pregnant patients on cervical polyp management. Herein, we propose an algorithm to guide patient counseling and cervical polyp management during pregnancy (Figure 4).

In asymptomatic patients with benign-appearing polyps, a conservative approach is usually preferred. In symptomatic patients, instead of proceeding with routine polyp removal as in nonpregnant patients, the risks of removal need to be balanced with benefits. For proper risk stratification, it is crucial to differentiate the type of polyp in addition to the physical characteristics of size, appearance, and location. In women with vaginal bleeding, ultrasound is essential for identifying possible alternate causes for antepartum bleeding such as threatened miscarriage, placental abruption, or placenta previa, in which cases polypectomy would not be the proper treatment. Even the incidental diagnosis of polyps can heighten women’s anxiety levels and result in further stress, thus necessitating further evaluation and counseling. 

## Conclusions

Ultrasound is particularly useful for differentiating between endocervical polyps and decidual polyps. Patients need to be counseled about the increased risk of spontaneous abortion and preterm delivery with the removal of decidual polyps versus endocervical polyps. The severity of symptoms and clinical risk factors should guide the need for removal in patients with decidual polyps given their associated morbidity. Rapidly growing polyps should raise suspicion of malignancy and may require further evaluation with colposcopy, oncology referral, and removal.

Future studies should stratify pregnancy outcomes based on polyp type. Additionally, future research should elucidate if the polyp or the polypectomy procedure is associated with chorioamnionitis, preterm labor, and spontaneous abortion. We believe that polypectomy may be safely performed in pregnant patients with endocervical polyps. Differentiating decidual and endocervical polyps via ultrasound is critical for patient counseling and developing safe management guidelines.
